# Navigating sex and sex roles: deciphering sex-biased gene expression in a species with sex-role reversal (*Syngnathus typhle*)

**DOI:** 10.1098/rsos.231620

**Published:** 2024-04-03

**Authors:** Freya A. Pappert, Arseny Dubin, Guillermo G. Torres, Olivia Roth

**Affiliations:** ^1^ Marine Evolutionary Biology, Zoological Institute, Christian-Albrechts-Universität Kiel, Kiel 24118, Germany; ^2^ Evolutionary Ecology of Marine Fishes, Helmholtz-Centre for Ocean Research Kiel (GEOMAR), Kiel 24105, Germany; ^3^ Institute of Clinical Molecular Biology (IKMB), University Hospital Schleswig-Holstein, Kiel University, Kiel 24105, Germany

**Keywords:** sex-biased gene expression, sex-role reversal, parental investment, male pregnancy, life-history trade-offs

## Abstract

Sexual dimorphism, the divergence in morphological traits between males and females of the same species, is often accompanied by sex-biased gene expression. However, the majority of research has focused on species with conventional sex roles, where females have the highest energy burden with both egg production and parental care, neglecting the diversity of reproductive roles found in nature. We investigated sex-biased gene expression in *Syngnathus typhle*, a sex-role reversed species with male pregnancy, allowing us to separate two female traits: egg production and parental care. Using RNA sequencing, we examined gene expression across organs (brain, head kidney and gonads) at various life stages, encompassing differences in age, sex and reproductive status. While some gene groups were more strongly associated with sex roles, such as stress resistance and immune defence, others were driven by biological sex, such as energy and lipid storage regulation in an organ- and age-specific manner. By investigating how genes regulate and are regulated by changing reproductive roles and resource allocation in a model system with an unconventional life-history strategy, we aim to better understand the importance of sex and sex role in regulating gene expression patterns, broadening the scope of this discussion to encompass a wide range of organisms.

## 1. Introduction

Males and females of the same species often display sexual dimorphism in morphological traits [1] stemming from sex-biased gene expression [2]. Based on which sex displays higher expression levels, genes are categorized as male-biased or female-biased [2]. The evolution of sex-biased gene expression can resolve conflicts between males and females, including sexual selection, sexual antagonism, relaxed selective constraint and conflicts in parental investment [1,2]. The prevalence of sex-biased gene expression is notable across numerous species, although its variation is assigned to differences across tissues and developmental stages [1–3], known to mostly be lowest during embryonic phases and highest in sexually mature adult stages [4].

Sex-specific gene expression has predominantly been investigated in species characterized by conventional sex roles, encompassing male–male competition and female parental care [1]. Given the evolutionary flexibility of sex roles and their variation among species and across an individual´s lifetime, research going beyond conventional sex roles is tremendously required for understanding the evolution life-history strategies [5–7]. Paternal care is the predominant parental care strategy in teleosts encompassing egg guarding and oral brooding to full viviparity [7,8]. The most devoted male parents can be accredited to the syngnathid family (seahorses, pipefishes and seadragons), which have evolved the unique male pregnancy ranging from simple attachment of eggs to the male ventral side to highly specialized brooding structures, analogous to those found in eutherian mammals, that provide embryos with protection, nutrients, oxygen and immunological components [8–12].

Experimental studies on parental investment, however, have traditionally focused on mothers' fitness, as in classical sex roles, they invest more in egg production and nurturing offspring, directly impacting the offspring’s fitness [13]. Notably, in some syngnathid species, research has expanded to explore the father’s fitness and investment in offspring, reflecting a broader understanding of parental roles [10,14–17]. The wide diversity of natural sex roles ranging from female-biased parental care with male mating competition to male-biased parental care with strong female competition offers opportunities to explore resource allocation trade-offs as drivers for sex-specific life-history strategy [18]. Life-history strategy evolved with distinct energy investment into somatic maintenance and reproduction, resulting in differences sex-specific resource allocation for immune defence, metabolism and longevity [19–21]. Unfortunately, we are lacking a comprehension of how patterns of sex-biased gene expression fluctuate in species featuring sex-role reversal with parental male-biased care. This basic research is crucial because gender dynamics are changing in human society and evolving over time. Currently, the concept of targeted medicine for sex-specific illnesses primarily relies on the binary classification of biological sex (egg or sperm producer) and tends to overlook the societal and environmental context of that sex. This approach creates a blind spot in biomedical research, as it fails to account for the costs of parental care and gender-specific settings that can result in shifts in gene expression.

To understand the importance of biological sex versus sex role in driving gene expression patterns, we compared sex-biased gene expression of young and older broad-nosed pipefish *Syngnathus typhle*. The syngnathid family with their sex-role reversal and their unique male pregnancy evolution facilitates decoupling the role of the female sex (defined as the contribution of eggs) and pregnancy (body reshaping and energy allocation) [11]. In the broad-nosed pipefish, the females produce on average more eggs than males can brood [16,22], resulting in males being the more reproductively constrained sex [23]. Larger brood size and limited resource availability influence the cost of male care, making embryo mortality inversely proportional to male condition (male mortality increases with a larger brood size) [12]. Given their polygamous mating system and males being the limited sex, males result in being the choosier sex, preferring larger females, whereas females are ornamented and actively court males [14,22–25]. In the present study, we sought to investigate how these sex-specific expression patterns vary between young and older pipefish individuals and unravel the influence of age and resource allocation shifts on sex-biased gene expression across distinct organs and conditions. We hypothesized that, owing to the sex-role reversal in *S. typhle*, there would be a reversal in patterns of sex-biased gene expression compared with conventional model species. This shift is anticipated to be more strongly associated with sex roles (i.e. parental investment, mate competition) rather than biological sex (i.e. egg or sperm producer) alone, and our expectation is that this reversed pattern would persist or even strengthen with increasing age. To test this, we conducted full transcriptome RNA sequencing (RNA-Seq) to analyse gene expression in three distinct organs: brain, head kidney and gonads, across different age groups (approximately 1 year old and over 2 years old) and between female and pregnant/non-pregnant male pipefish. Previous research has shown significant differences in sex-biased gene expression across organs, with gonads showing the most pronounced variations [1,26]. We chose to include the brain and head kidney because of their more similar development in both males and females. Furthermore, in teleost fish, the head kidney plays a vital role in immune regulation, making it an important focus for studying sexual differences in gene expression related to immunity [27]. Our findings deepen our understanding of this unique system and contribute to discussions about sexual dimorphism and parental investment as drivers of gene expression.

## 2. Methods

### 2.1. Sample collection

Broad-nosed pipefish *S. typhle* were caught in the southwestern Baltic Sea (54°39′ N, 10°19′ E) in spring, right before their breeding season [28]. They were brought to the GEOMAR institute, where body mass and total body length were measured. In our study, we used 10 females and 20 males; the latter included 10 pregnant and 10 non-pregnant. We also separated the pipefish based on age, *ca* 1 year old for young pipefish and above 2 years for old (their estimated lifespan in the wild is 3 years) and measured body size and weight as additional means of assessment (electronic supplementary material, figure S1). We acknowledge the potential limitations of this approach as variability in growth rates and environmental factors may lead to misclassifications, due to fast- or slow-growing individuals. However, given the limited availability of established models for studying non-model organisms like pipefish, our approach draws on years of fieldwork experience. Broad-nosed pipefish engage in mating and breeding activities during spring, followed by migration to unknown areas during winter, before returning to the seagrass meadows of the southwestern Baltic Sea for breeding in the subsequent year [28]. The offspring cohorts from one breeding season typically only reach sexual maturation during the next breeding season, indicated in males over the development of a brood pouch that only becomes visible during the next breeding season (phenotypic signal for sexual maturation). This resulted in five individuals for each group young male (YM), young pregnant (YP), old male (OM), old pregnant (OP), young female (YF) and old female (OF).

The animals were sacrificed with an overdose of MS-222 (tricaine methane sulfonate, 500 mg l^−1^; Sigma-Aldrich). Carcasses were dissected, and different organs including brain, head kidney and gonads were sampled and immediately preserved in RNA later. The samples were kept at 4°C for 3 days, before being transferred to −20°C for long-term storage. Previous studies have highlighted substantial variability in sex-biased gene expression among organs [26,29,30]. Gonads exhibit the highest degree of sex-biased expression, with most biased genes involved in sex differentiation and fertility regulation [1,26]. Notably, brain-specific dimorphism has demonstrated preliminary trends in *S. scovelli* [31], while the head kidney—a unique organ in teleost fish analogous to the adrenal gland—serves the dual function of producing red and white blood cells, thereby supporting both oxygen transport and immune defence [27]. Given its prominent role in immune regulation, the head kidney becomes a focal point for studying genes displaying sexual immune dimorphism in expression as identified in diverse species from insects to lizards, birds and mammals, with an overall stronger innate and adaptive immune response in females [32,33].

### 2.2. RNA isolation and Illumina sequencing

For our RNA-Seq analysis, we extracted RNA from brain, head kidney, ovaries and testes. The RNA extraction was performed using RNeasy Mini Kit (Qiagen, Venlo, The Netherlands) according to the manufacturer’s protocol. Extraction yields were quantified using NanoDrop ND-1000 spectral photometer (Peqlab, Erlangen, Germany), RNA was then stored at −80°C (for information on quality and quantity of RNA for RNA-Seq refer to electronic supplementary material, table S5). Library preparation (TruSeq stranded mRNA Kit by Illumina) and sequencing (RNA-Seq NovaSeq6000 S4, 2 × 150 bp, over 17M clean paired-end reads per sample) were carried out at the Institute of Clinical Molecular Biology (IKMB).

### 2.3. Data analysis

#### 2.3.1. Morphological analysis

Statistical analysis was done in Rstudio (v. 4.2.2) [34], and tests were considered significant when *p*-values were smaller than *p* = 0.05. Mean and standard deviations were calculated, and in a separate two-way ANOVA, the differences between weight (g) and length (cm) for sex and age were analysed, with Tukey’s honestly significant difference (TukeyHSD) *post hoc* test for single interactions (boxplots for weight and length: electronic supplementary material, figure S1).

Two samples had to be excluded due to sequencing failure (one YM testes and one OM pregnant testes). The resulting reads were quality-controlled using FastQC v. 0.11.9 [35] and trimmed using Fastp v. 0.20.1 [36]. Reads were aligned to a whole genome assembly of *S. typhle* (BioProject ID: PRJNA947442) using STAR v. 2.7.9a [37]. Transcript abundance was quantified with TPM Calculator [38].

Statistical analysis in Rstudio (v. 4.2.2) [34] used the edgeR package (v. 3.40.2) [39]. We scaled the raw count data with 26 072 genes to counts per million (cpm) and filtered it based on a minimum threshold of 10 counts in at least 20 libraries, resulting in 19 348 genes. To control for composition biases, we normalized the data using the trimmed mean of values (TMM) with the *calcNormFactors* function.

**Figure 1.**
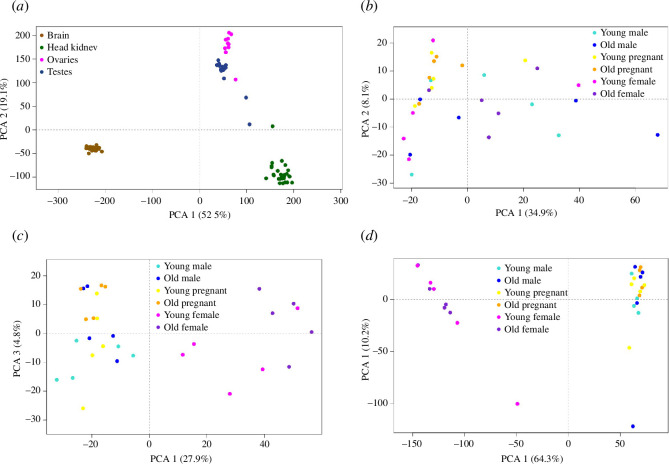
PCA of gene expression patterns. (*a*) PCA plot illustrating sample clustering based on organ type. Each organ is represented by distinctive colours: brain in brown, head kidney in green, ovaries in magenta and testes in dark blue. Subsequent PCA plots provide detailed organ-specific analyses, incorporating distinctions for sex and life stage: young males in light blue, old males in blue, young pregnant individuals in yellow, old pregnant individuals in orange, young females in pink and old females in purple. (*b*) PCA plot for the brain, revealing no discernible clustering for sex, age or pregnancy. (*c*) PCA plot for head kidney, focusing on PC1 and PC3. While PC1 clearly shows separation for sex, PC3 highlights an age effect with young individuals clustering more on the lower half and older individuals on the top half of the plot (additional insights into PC2 can be found in electronic supplementary material, figure S2). (*d*) PCA plot for gonads, depicting clear separation between ovaries and testes on PC1, with no discernible impact of life stages.

We initially performed a principal component analysis (PCA) with the ‘regularized log transformation procedure’ (rld) transformed expression values across all the samples to validate organ separation and check for outliers (figure 1 and electronic supplementary material, figure S2). Once we had assessed that organ type explained most variation in the dataset, we focused on single organ’s differential gene expression (DGE) analysis, to detect age, sex and pregnancy effects. The raw count data were separated in brain, head kidney, ovaries and testes. We reapplied filtering and normalization steps and resulted in 18 373 genes in brain, 16 746 in head kidney, 16 368 in ovaries and 18 218 in testes. For each organ, we plotted PCAs (figure 1) and tested our hypotheses using a permutational multivariate analysis of variance using distance matrices (PERMANOVA) using function ‘adonis2’, method ‘bray’ and 999 permutations (adonis2 (counts ~ meta$age*meta$sex*meta$pregnant, permutations = 999, method = ‘bray’). As a *post hoc* we used the *pairwise.adonis* function with Bonferroni correction.

Subsequently, we used the *limma* package (v. 3.54.1) for conducting DGE analysis [40]. *limma* employs a linear model approach coupled with empirical Bayes techniques and uses the Voom method to transform count data into a continuous scale. This method is especially effective in addressing batch effects and reducing technical variability. The Voom method is specifically adept at handling varying library sizes [41]. To compare multiple groups (electronic supplementary material, table S2), we constructed a matrix containing independent contrasts. This matrix facilitated conducting a one-way analysis of deviance (ANODEV) for each gene. Following this, we calculated coefficients representing differences between groups to ascertain log fold changes (logFC) through the application of the *contrasts.fit* function. Then, we implemented empirical Bayes moderation to shrink the estimated variance and conducted moderated *t*-tests to pinpoint genes with significant differential expression. For subsequent downstream analysis, our focus was exclusively on adjusted *p*-values below 0.05 and logFC ±1. This ensured that the observed effects of treatments were statistically meaningful.

#### 2.3.2. Gene ontology and enrichment analysis

For gene ontology (GO) analysis, we annotated the differentially expressed genes (DEGs) using a homology-based search of *Danio rerio* (GRCz11) with OrthoFinder [42]. *Danio rerio* orthologues from the resulting DEGs between YM versus YF and OM versus OF in head kidney were uploaded to g:Profiler web tool (last accessed on 11 August 2023) [43] to perform gene enrichment analysis.

The settings used were Organism ‘*Danio rerio* (Zebrafish)’, statistical domain scope set at ‘All known genes’, significance threshold ‘g:SCS threshold’ at 0.01, numeric IDs treated as ‘ENTREZGENE_ACC’, the data sources were limited to GO biological processes and biological pathways the databases Kyoto Encyclopedia of Genes and Genomes (KEGG) and Reactome (REAC) (electronic supplementary material, tables S4). To make our analysis more specific, we applied a maximum term size of 1000 to exclude overly broad categories and prioritize more informative outcomes.

## 3. Results

### 3.1. Sex-biased expression between organs

The results illustrating the variations across distinct organs are depicted in the PCA plot shown in figure 1*a*. The samples displayed strong clustering based on their organ types, and the brain exhibited the most distinct separation compared with the other organs. Given that the primary source of variance within the dataset arose from organ types, and our interest was focused on delving into the impacts of sex, age and pregnancy status, we proceeded to conduct a more granular analysis within each individual organ.

Upon closer examination of the brain, hypothesis testing did not reveal any statistically significant disparities for the studied variables or their interactions (PERMANOVA, *p* > 0.05). This outcome is also visually evident in the PCA plot specifically dedicated to brain tissue (figure 1*b*). While discernible differences did exist among the sex-specific gonadal tissues (figure 1*d*), a detailed exploration within the testes and ovaries of the two distinct age groups (young versus old) failed to unearth any noteworthy effects. Nonetheless, a modest effect emerged from the interaction between age and pregnancy status (yes or no) within the context of testes (PERMANOVA, *p* = 0.034). Subsequent pairwise comparisons, however, did not yield any interactions that reached a statistically significant threshold. In terms of head kidney, neither age nor pregnancy displayed substantial impacts, whereas sex exerted a highly significant influence (PERMANOVA, *p* = 0.001). This distinction is depicted in the PCA plot presented in figure 1*c*, wherein PC1 accounts for approximately 28% of the variance between samples. Additionally, PC3 appears to differentiate between young and old individuals, with both OM and OP subjects clustering towards the upper left corner, and OF individuals gravitating in a similar direction on the right (figure 1*c*). Despite its relatively small variance of 4.8%, a two-way ANOVA for PC3 (age and sex) unveiled a significant effect of age (*p* = 0.0014).

The differential gene expression analysis suggested that distinctions pertaining to sex and age were particularly pronounced within the head kidney (electronic supplementary material, tables S2 and S3). Notably, in the contrast comparison between YM and YF, 1930 significant DEGs were identified (adj. *p* < 0.05), while the comparison between OM and OF yielded 3564 DEGs. Nonetheless, no DEGs were observed when comparing pregnant and non-pregnant males across all organs (electronic supplementary material, table S2). Results in the brain revealed only a couple of DEGs, which were discarded later in the analysis owing to either low logFC or the absence of *Danio rerio* annotations (electronic supplementary material, tables S2 and S3).

**Figure 2.**
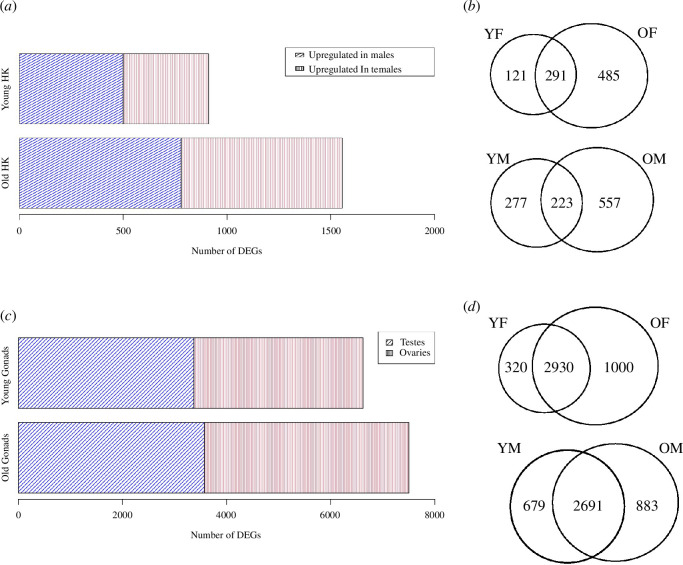
This figure presents the outcomes of the differential gene expression analysis in the head kidney (adj. *p* < 0.05; logFC ±1), focusing on young males (YM) vs. young females (YF), as well as old males (OM) versus old females (OF). (*a*) DEGs between male and female in head kidney. Bar plots illustrate the genes that exhibit upregulation in males compared with females, stratified across different age groups for head kidney. YM display overall 500 upregulated genes versus 412 in YF, while OM showed 780 upregulated genes versus 776 in OF. (*b*) Venn diagram showing overlapping sets of DEGs between YF and OF, as well as between YM and OM. This helps us discern genes that exhibit sex-specific biased expression irrespective of age and those whose sex-biased expression changes particularly with age. (*c*) DEGs between male and female in gonadal tissues. Bar plots showing differential gene expression between testes and ovaries, for old and young pipefish. There were 3370 genes biased in YM testes versus 3250 in YF ovaries, and 3574 in OMs versus 3930 in OF. (*d*) Venn diagram showing overlapping sets of DEGs between males and females between young and old. Majority of DEGs are sex-biased irrespective of age.

Conversely, differential gene expression analysis within the respective gonadal organs (testes and ovaries) displayed an absence of significant DEGs, when considering age or pregnancy (electronic supplementary material, table S2). Yet, analysis of testes versus ovaries revealed a high number of significant DEGs for age (electronic supplementary material, table S2). With a logFC threshold of ±1, we found 6620 DEGs between YM versus YF, with 3370 upregulated in YMs and 3250 in YFs, and 7504 for OM versus OF, with 3574 genes upregulated in OMs and 3930 upregulated in OFs (figure 2*c*). However, when checking for overlaps of DEGs between the two age groups, we found that the majority of DEGs did not change with increasing age (figure 2*d*). Meaning that most genes were sex-biased irrespective of increasing age. Nonetheless, there were roughly 999 significant DEGs between YMs and YFs which were not differentially expressed between OM and OF and 1883 vice versa, making these age-dependent (figure 2*d*).

**Figure 3.**
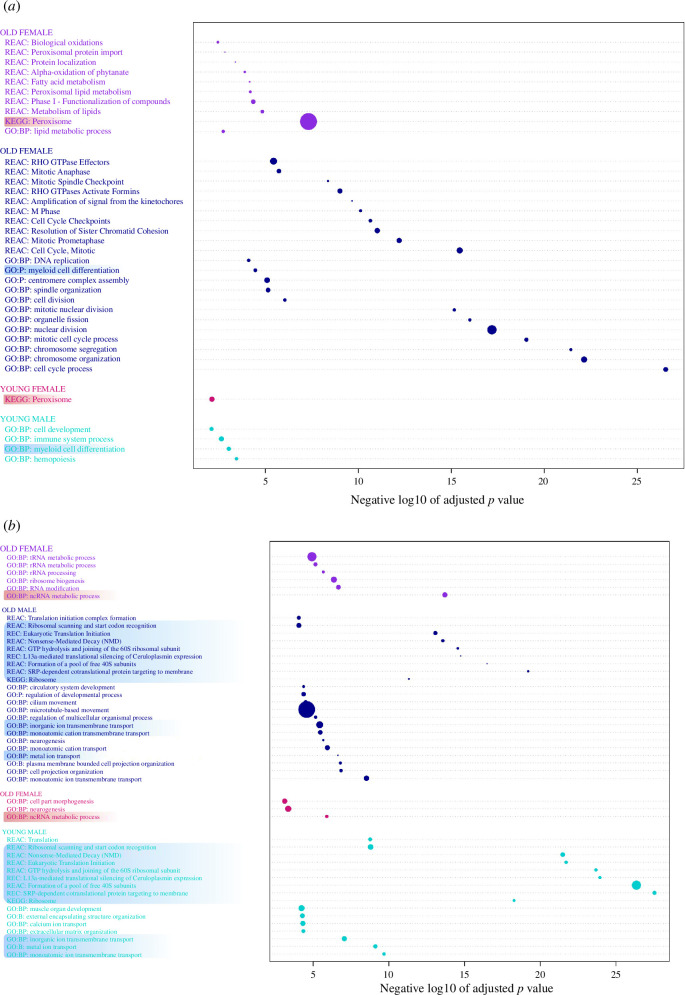
Gene set enrichment analysis results for *Danio rerio* annotated DEGs (logFC ±DE1, adj. *p*l < 0.05). Results are for (*a*) GSEA results head kidney and (*b*) GSEA results gonads. Purple colour represents pathways enriched for sex-biased expressed genes in OF pipefish, dark blue for OM, pink for YF and light blue for YM. Highlights reflect pathways that were enriched irrespective of age, in light red we match YF and OF, and in light blue YM and OM. This is only within the respective organs. Data sources were limited to GO biological pathways, KEGG and Reactome, with a g:SCS threshold of 0.01. *X*-axis shows the negative log10 of the adjusted *p*-values. Dots are scaled to reflect proportions of genes within the specific category. As there were numerous pathways highly enriched in OMs, we set a more stringent threshold of adj. *p* < 0.0001 and removed repetitive pathways for easier reading (full results are found in electronic supplementary material, table S4).

Figure 3*b* displays the most enriched pathways for gonads in different age groups, highlighting the pathways consistently biased by sex regardless of age. This encompasses overlapping genes from figure 2*d* as well. There was a significant overlap between young and old males (figure 3*b* and electronic supplementary material, table S4), including translation initiation, metal ion transport and nonsense-mediated decay (NMD), and genes encoding for both tissue inhibitors of metalloproteinases (TIMP) and a disintegrin and metalloproteinase (ADAM) metallopeptidase, metalloproteinase, along with various types of ribosomal protein-encoding genes (electronic supplementary material, table S3, gonads). In ovaries, we observed pathways enriched for ncRNA metabolic processes and several pathways related to RNA modification in older females' ovaries (figure 3*b* and electronic supplementary material, table S4). The involved genes mostly encompass tRNA methyltransferases and zinc fingers (electronic supplementary material, table S3, gonads).

### 3.2. Sex-biased gene expression in head kidney

Results from the differential gene expression analysis in head kidney revealed 912 significant DEGs (with logFC threshold of ±1) in the contrast comparison between YM and YF, and 1556 DEGs between OM and OF (figure 2*a*). In the first case, 500 genes exhibited upregulation in YM and 412 in YF, while for the OM versus OF comparison, 780 were upregulated in OM and 776 in OF. Overlaps between sex-biased expressed genes for young and old pipefish found that 291 DEGs were upregulated in both YF and OF, whereas approximately 223 were the same for both YM and OM (figure 2*b*). Gene set enrichment analysis (GSEA) on the latter revealed that female-biased genes, regardless of age, displayed enrichment (*p* < 0.01) in processes such as transport, peroxisome and the terminal pathway of complement (figure 3*a* and electronic supplementary material, table S4). In contrast, male-biased genes exhibited enrichment in myeloid cell differentiation and haemopoiesis (figure 3*a* and electronic supplementary material, table S4). Older females exhibited enrichment across categories linked to diverse metabolic pathways, encompassing lipid metabolism, fatty-acid metabolism and peroxisomal lipid metabolism (figure 3*a* and electronic supplementary material, table S4). Furthermore, heightened activities in cell respiration and oxidation processes, exemplified by pathways such as peroxisome, alpha-oxidation of phytanate and biological oxidations (figure 3*a*). In contrast, YF predominantly exhibited enrichment in the peroxisome pathway (figure 3*a*).

Genes upregulated in OF compared with OM that were integral to metabolic pathways encompassed several cytochrome P450 enzymes such as *cyp1b1, cyp2r1, cyp4v7, cyp8b1* and *cyp19a1a*, constituting a superfamily of enzymes pivotal in metabolizing a spectrum of compounds, including drugs, toxins and hormones [44]. CYP1 localizes to the mitochondrial inner membrane, catalysing the critical initial step in steroid biosynthesis, the conversion of cholesterol to pregnenolone hormones [44]. CYP2 enzymes, as recognized in mammals, metabolize vitamin D and arachidonic acid hormones [44]. While CYP19, also known as aromatase, facilitates the conversion of C19 androgen to aromatic C18 oestrogen [44]. Further delving into lipid metabolism, several female-biased expressed genes stood out, including fatty acid amide hydrolase (*faah*), lipoprotein lipase (*lpl*) and apolipoprotein L 1 (*apol1*), A-II (*apoa2*) and apolipoprotein C-II (*apoc2*). Notably, *faah, apoc2* and *lpl* also exhibited upregulation in YFs compared with YMs (electronic supplementary material, table S3). The peroxisome pathway displayed female-biased enrichment in both YF and OF (figure 3*a*). These enzymes play pivotal roles in fatty acid oxidation and lipid metabolism at large, while also contributing to the detoxification of harmful compounds, including reactive oxygen species (ROS) [45]. Particularly pronounced in OF were genes related to cell respiration and oxidation, such as various glutathione peroxidase forms (*4a* and *4b*) and peroxiredoxin (*prdx1*, also observed in YF). Additionally, genes involved in respiration NADPH oxidase 1 (*nox1*) and 4 (*nox4*), acyl-CoA oxidase 3 (*acox3*, also evident in YF), ferredoxin reductase (*fdxr*) and ferredoxin 1 (*fdx1*, also observed in YF) exhibited upregulation (electronic supplementary material, table S3).

Numerous pathways exhibited enrichment in OMs relating to cell cycle, chromosome organization, DNA replication and assorted cellular processes (figure 3*b* and electronic supplementary material, table S4). Some DEGs that overlapped between these pathways belonged to the kinesin family (*kifc1, kif11, kif14, kif15, kif20a, kif20ba, kif22, kif23* and *kif18a*) or centromere proteins (*cenpn, cenpe, penpk, cenpo, cenph, cenpt, cenpf* and numerous others) (electronic supplementary material, table S3). Additionally, still in OM pipefish, pathways concerning DNA repair (GO:0006281, *p* < 0.002) and DNA damage response (GO:0006974, *p* < 0.0001) exhibited enrichment (electronic supplementary material, table S4). Notable genes within these pathways encompass damage-specific DNA binding protein 2 (*ddb2*) and DNA repair contributors *rad51b* and *rad54*, which play specific roles in homologous recombination-based DNA repair processes [46]. Furthermore, ATM serine/threonine kinase (*atm*), checkpoint kinase 1 (*check1*) and checkpoint kinase 2 (*chek2*), with their overarching responsibilities in activating downstream targets implicated in DNA repair and cancer prevention [47,48], were also represented.

Importantly, the timeless circadian clock gene exhibited upregulation in both young and old males compared with females (electronic supplementary material, table S3). Although fish-specific research in this context is lacking, studies in humans have revealed that its downregulation leads to telomere-associated DNA damage [49,49]. Notably, intriguing male-biased expressed genes included *deptor*, a negative regulator of the mammalian target of rapamycin (mTOR) signalling pathway that acts as a feedback inhibitor [50]. Additionally, insulin-like growth factor 2 mRNA binding protein 2a (*igf2bp2a*) and, in older males, peroxisome proliferator-activated receptor gamma, coactivator 1 alpha (*ppargc1a*), a homologue of forkhead box O (*FOXO*) 1, all interconnected in nutrient sensing pathways and collectively influential in cell growth, metabolism and lifespan regulation [51,52].

For genes particularly upregulated in YMs compared with YFs, we observed enrichment in haemopoiesis, cell development and myeloid cell differentiation, although these also exhibited enrichment in OMs relative to females albeit to a lesser extent (figure 3*a* and electronic supplementary material, table S4). These pathways' enrichment make sense, given the head kidneys’ role in producing red and white blood cells [27]. Interestingly, YMs uniquely exhibited enriched genes associated with immune system processes (figure 3*a* and electronic supplementary material, table S4). Within this pathway genes included chemokine receptors 1 (*cxcr1*) and 4a (*cxcr4a*), interferon regulatory factor 5 (*irf5*) and toll-like receptor 8 (*tlr8*), the latter being a homologue of human *tlr1* [53,54]. There was also V-set immunoregulatory receptor (*vsir*), analogous to other immune checkpoint molecules, VSIR (VISTA) plays a role in modulating T-cell responses, acting as a negative regulator of T-cell activation to prevent excessive immune responses and maintain immune homeostasis [55,56]. Additionally, the gene cluster of differentiation (CD) antigens, such as *cd7al* and *cd247l*, were identified, which participate in immune recognition processes [57]. Notably, recombination activating gene 1 (*rag1*) was also found upregulated in YMs, encoding a protein crucial for V(variable), D(diversity), and J(joining) (V(D)J) recombination, this process is fundamental for generating diverse antigen receptor molecules on immune cells, specifically B and T cells [58,59]. Moreover, we observed pro-inflammatory cytokines expressed in males, including neutrophil cytosolic factors (*ncf1, ncf2* and *ncf4*), where NCF1 stands out as a pro-inflammatory protein contributing to the generation of reactive oxygen species that are pivotal for pathogen eradication [60].

Although females did not show enrichment in immune pathways, upon closer observation, we found several immune-related genes notably upregulated in both young and old females compared with males (electronic supplementary material, table S3). These included interleukins and their receptors, such as *il11ra*, *il17ra1a*, *il19l* and *il10*, the latter suppresses the production of pro-inflammatory cytokines such as TNF-alpha [61], and a major histocompatibility complex class I ZCA (*mhc1zca*) (electronic supplementary material, table S3), which are molecules present on the surface of all nucleated cells and bind to foreign antigens so that CD8+ T cells can recognize and eliminate the invading pathogens [62]. Furthermore, interleukin 6 signal transducer (*il6st*) and interferon regulatory factor 6 (*irf6*) were elevated in YFs.

## 4. Discussion

Our work presents a unique exploration into the intricate relationship between male and female life-history traits and their influence on gene expression patterns, particularly in the context of a sex-role reversed species featuring male pregnancy, *S. typhle*. By conducting comparative analyses of gene expression among males, females, young, old, pregnant and non-pregnant male pipefish across various organs (brain, head kidney, gonads), our aim was to uncover fresh insights into the roles of sex (i.e. egg or sperm producer) and sex role (i.e. parental investment, mate competition) in shaping sex-biased gene expression patterns.

Our findings highlighted distinct gene expression patterns across different organs, whole brain, head kidney and gonads. The most abundant DEGs were observed in the gonads and head kidney, with an occurrence of more sex-biased expressed genes between older pipefish, which aligns with research findings in other organisms [4]. While examining the brain, no substantial evidence of sex differences emerged, aligning with findings in various teleost species, including East African cichlids [29], sparid sharpsnout seabream (*Diplodus puntazzo*) [30], the bluehead wrasse (*Thalassoma bifasciatum*) [63] and in the pipefish species *S. scovelli* [31], where only a limited number of sex-biased genes were detected. It is essential, however, to acknowledge the potential variability in sex-biased gene expression across species, as illustrated by studies in the tropical gar (*Atractosteus tropicus*) [64] and zebrafish [65]. This emphasizes the significance of recognizing species-specific nuances within our study, providing valuable insights into the diversity of sex-related gene expression patterns in the teleost brain. Interestingly, pregnancy exhibited minimal or no effect on gene expression across all examined organs. This observation suggests that the physiological changes associated with pregnancy may not significantly affect the selected organs, considering that another study comparing pouch tissues and pregnancy gradients across four different syngnathid species found DEGs in immune pathways and metabolic processes [66,67].

**Figure 4.**
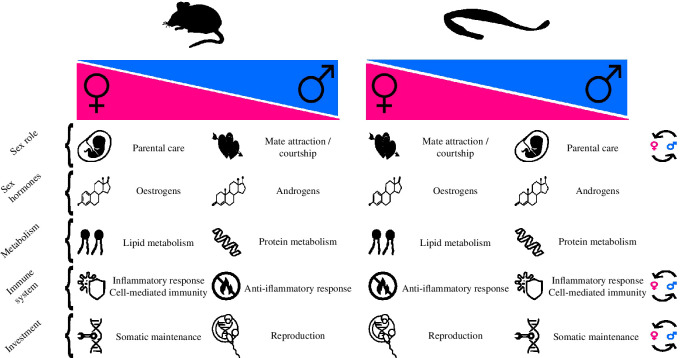
This section provides a summary comparison of traits between a conventional sex-role mouse model and the broad-nosed pipefish, which displays sex-role reversal. The diagram highlights the differences in these traits based on biological sex (magenta for females and blue for males). Circular arrows on the far right indicate potential reversals in life-history traits compared with the conventional model species, based on known literature (sex role [24,25,66], sex hormones [68,69] and immune system [11,67,70]) and results presented in this paper (including metabolism, immune system and investment). The relevant literature supporting key points in mammals includes references for sex roles [71], sex hormones [72], metabolism [73,74], immune system [33,75] and investment [73,76].

Based on the results and the reproductive biology of *S. typhle*, we suggest that female pipefish may allocate a relatively higher proportion of their resources to pre-copulatory reproduction compared with males. This inference is drawn from the enrichment of female-biased pathways related to RNA modification in ovaries and the observation that older females display enrichment in metabolic pathways associated with lipid and fatty-acid metabolism in the head kidney (figure 3). These patterns suggest a potential emphasis on oocyte development, maturation and hormonal regulation, indicating a notable allocation of resources, possibly towards pre-copulatory investment encompassing egg mass and physiological growth to attract more mates (figure 4) [14].

The gonads of male pipefish also revealed pathways associated with sperm quality and ejaculate volume such as metal ion or calcium transport (figure 3*b*), including metallopeptidase and metalloproteinase genes (electronic supplementary material, table S3, gonads) [77]. However, there were not many other pathways specifically related to generating sperm and associated fluids/proteins. These results are consistent with research in *S. scovelli*, where a reduced expression of genes associated with sperm function was found, suggesting that pipefish may have evolved to produce a smaller number of sperm [77]. The study also suggests that due to the selection for male pregnancy, this could potentially lead to a decrease in sperm count and shift energetic demands towards developing the brood pouch itself. Interestingly, the enriched NMD pathway also exhibited numerous male-biased genes in testes (figure 3*b*). This pathway, crucial for RNA quality control and precise gene expression regulation, may play a vital role in maintaining the integrity of spermatogenesis [78].

In the head kidney, female-biased genes, irrespective of age, exhibited a notable enrichment primarily in the peroxisome pathway. These membrane-bound organelles play pivotal roles in lipid metabolism within cells, maintaining cellular oxidative equilibrium [79]. Given the energy investment in processes like oocyte development by female pipefish, the heightened peroxisomal pathway enrichment suggests an augmented demand for lipid metabolism to fuel these activities. Notably, older females displayed enrichment in metabolic pathways pertinent to lipid and fatty-acid metabolism (in head kidney), including peroxisomal lipid metabolism. Enriched genes within these pathways, such as *faah* [80], *apolipoproteins* [81] and *lpl* [82], collectively influence lipid metabolism, lipase activity and energy production. The key role of LPL, as a rate-determining enzyme for fatty acid supply to various tissues, hints at its potential to govern the allocation of dietary lipids towards storage or utilization [82–84].

Regulating dietary fats holds critical importance in fish, serving as both an energy reservoir and a fundamental source of essential fatty acids (EFAs) vital for growth, development and reproduction [83,85]. While basic metabolic processes align between males and females, variations in body composition, sex hormones, sex-specific energy expenditure and age collectively influence nutrient metabolism and energy storage efficacy [73,86]. Notably, oestrogen concentrations influence fat deposition and storage patterns in the body, aligning with findings in *S. typhle* where males produce testosterone and females produce oestrogen despite the reversed reproductive roles [68,87]. The involvement of multiple cytochrome P450 enzymes, particularly OFs, such as CYP1 enzyme catalysing the conversion of cholesterol to pregnenolone hormones, which is the starting point in the production of cortisol, oestrogen, progesterone and testosterone [44], and CYP19 helping the conversion of androgen to aromatic oestrogen (oestrone and 17β-oestradiol) [44], implies potential links to oestrogen-driven processes. Thus, the interplay between energy allocation to eggs and ovarian oestrogen production likely contributes to the sex-biased expression of lipid metabolic-related genes [88]. Considering the hormonal pathways inherent to females, an equally valid alternative hypothesis could be that the increase in lipid and fatty acid metabolism may be due to a greater emphasis on fat storage in females compared with males. This would be consistent with the generally larger body size of female individuals within the species, highlighting the complex connection between hormonal control, energy allocation and reproductive strategies [23,25,89].

In contrast, our results suggest that male pipefish, especially older individuals, may exhibit a strategic investment in their somatic maintenance. In the head kidney of OMs, we found pathways pertaining to DNA repair and damage response, indicating potentially a more robust DNA repair machinery in older males. Male-biased genes, such as *deptor, igf2bp2a* and *ppargc1a*, known for nutrient sensing and pathways related to mTOR, insulin-IGF regulation and FOXO transcription factors, suggest a focus on cellular maintenance, stress resistance, maintenance of cellular homeostasis and, potentially, longevity [50–52]. The presence of the *timeless circadian clock* gene, associated with genomic integrity and potential telomere-associated maintenance [46], further reinforces the idea of male investment in health. Together, these findings may reflect male pipefish investing in mechanisms that promote their own health and somatic well-being (figure 4).

An intriguing result was the heightened enrichment of processes related to haematopoiesis and the immune system in YMs as compared with YFs, encompassing genes linked to chemokine receptors, interferon regulatory factors, toll-like receptors and immune checkpoint molecules. Considering that male pipefish undergo pregnancy and harbour embryos in their pouches, the increased emphasis on haematopoiesis and immune cell development in YMs might signify an evolutionary adaptation aimed at bolstering their own immune system [9]. There are inherent trade-offs associated with balancing the reproductive system with the immune system. In species with internal fertilization and gestation, such as pipefish, there are specific demands placed on the immune system, including the need to avoid sexually transmitted diseases and prevent rejection of the developing embryo [90,91]. Moreover, the upregulation of *rag1* is noteworthy, as this gene is pivotal for V(D)J recombination—a process generating diverse antigen receptor molecules on immune cells [59]. This broader repertoire of immune receptors could effectively recognize a wider array of pathogens. This divergence might reflect evolutionary adaptations that cater to distinct immune system demands between the two sexes [66]. We know that sex-specific pathogens exist in other fish species due to sex-specific behaviours, such as shoaling behaviour in female guppies' increased ectoparasite loads [92]. Preliminary in-house data suggest that there is a difference in ectoparasite prevalence between male and female pipefish and there is evidence to suggest that parent-specific transgenerational immune priming does occur in broad-nosed pipefish [9]. The increased number of YM-biased immune genes are in contrast to studies in mammals (figure 4), where it has been shown that cell-mediated immunity seems to be more pronounced in females with both dendritic cell antigen-presenting genes, TLRs and immunoglobulins proliferating at higher rates [33,93]. Furthermore, sex-hormone-specific research has shown that androgens typically induce an anti-inflammatory response [33,94], increasing also *il10* production, which conversely, was significantly upregulated in female pipefish (having higher oestrogen levels) in our dataset, with also numerous pro-inflammatory markers being upregulated in males (with higher testosterone; electronic supplementary material, table S3). The enriched immune pathway in YM might indicate a sex pattern reversal of inflammatory response compared with conventional sex-role species (figure 4), thus making pregnancy and the life-history trade-offs play a more significant role here. It was surprising, however, that we did not find the same immune system pathway enriched in OM and that there were fewer sex-biased immune genes between OM and OF. Ageing is associated with a decline in immune function, often referred to as immune senescence [95]. This decline tends to affect both males and females similarly and can lead to a more uniform immune response among older individuals [75,95]. This could in part explain our results, but further research is needed.

Our results illuminate that sex-biased gene expression is a dynamic interplay influenced by both biological sex and sex-specific life-history traits. Within this intricate framework, resource allocation trade-offs into parental care can exert profound impacts on the patterns of sex-biased gene expression. Notably, our study suggests that some genes may be more influenced by biological sex, while others are shaped by sex roles. While these findings provide insights into potential differences in resource allocation between the sexes, it is important to note that direct quantification of reproductive investment requires additional research, such as assessments of reproductive effort or energy allocation. Moreover, to gain a more holistic understanding of the molecular processes governing sex-specific gene expression, it is crucial to consider post-transcriptional events, including the influence of non-coding RNAs on protein synthesis. Our research reveals that the gene expression landscape undergoes significant changes with age. Males and females exhibit distinct gene expression profiles, with some pathways converging (e.g. in the pipefish immune system) and others diverging (e.g. in anti-ageing mechanisms) over time. These age-related shifts underscore the importance of studying sex-biased expression also in context of senescence and its distinct physiological changes between males and females. The interplay between biological sex, sex roles, age and organ-specific factors can yield diverse and context-dependent gene expression patterns. In future research, we aim to extend our research beyond a single species of syngnathid, given the diverse array of pregnancy gradients, sex roles and mating systems present within the family [24,96]. We hope to have underscored the importance of a nuanced approach to the study of sex-specific gene regulation, accounting for the multifaceted nature of sex and sex roles in shaping gene expression dynamics.

## Data Availability

Supplemental information include additional figures (electronic supplementary material, figures S1, S2), morphology measurements (electronic supplementary material, table S1), the significant differentially expressed genes (electronic supplementary material, tables S2, S3), the gene set enrichment analysis results as tables (electronic supplementary material, table S4) and information on quality and quantity of RNA for RNA-Seq (electronic supplementary material, table S5) [97]. The raw sequencing data and metadata used in this study is available from the National Center for Biotechnology Information (NCBI) Sequence Read Archive (SRA) under BioProject ID PRJNA943164 with submission ID SUB12940929. The whole genome assembly of *Syngnathus typhle* can be found under BioProject ID PRJNA947442 with submission ID SUB12974183.
